# Nutritional composition and antinutritional properties of maize ogi cofermented with pigeon pea

**DOI:** 10.1002/fsn3.571

**Published:** 2018-01-22

**Authors:** Uchechukwu I. Okafor, Adebunkola M. Omemu, Adewale O. Obadina, Mobolaji O. Bankole, Samuel A. O. Adeyeye

**Affiliations:** ^1^ Department of Microbiology Federal University of Agriculture Abeokuta Nigeria; ^2^ Department of Hospitality and Tourism Federal University of Agriculture Abeokuta Nigeria; ^3^ Department of Food Science & Technology Federal University of Agriculture Abeokuta Nigeria; ^4^ Department for Management of Science and Technology Development Ton Duc Thang University Ho Chi Minh City Vietnam

**Keywords:** antinutritional, maize, nutritional, ogi, pigeon pea

## Abstract

Maize was cofermented with pigeon pea for ogi production and evaluated for nutritional (proximate composition, minerals, vitamins, and amino acid profile analyses) and antinutritional (phytate, tannin, and trypsin inhibitor activity analyses) qualities. White maize and pigeon pea were mixed at ratios of 90:10, 80:20, 70:30, 60:40, and 50:50, respectively, with 100:0 serving as the control. Mixtures were cofermented for 96 hr at 27°C ± 2°C and nutritional, mineral, and antinutritional qualities were analyzed using analysis of variance. Results of proximate analysis showed that the values were significantly difference at *p *≤* *.05. Maize cofermented with pigeon pea at a ratio of 60:40 had the highest protein (22.79 mg/100 g), fat (19.27 mg/100 g), ash (2.98 mg/100 g), crude fiber (0.73 mg/100 g), and lowest moisture (1.98 mg/100 g) content, and was significantly (*p *≤* *.05) different from the other ratios. Of all the mixtures analyzed, 60:40 was significantly (*p *≤* *.05) different and had the highest Vitamin B_1_, B_2_, and B_3_ contents. Amino acid profile results showed that maize cofermented with pigeon pea at a ratio of 60:40 showed the highest contents of lysine (93.95 mg/g), tryptophan (20.38 mg/g), isoleucine (54.78 mg/g), phenylalanine (86.23 mg/g), leucine (109.55 mg/g), and valine (68.29 mg/g), respectively, and was significantly (*p *≤* *.05) different from the other ratios. Results of antinutritional analysis showed low phytate, tannin, and trypsin inhibitor values in maize cofermented with pigeon pea at a ratio of 60:40 when compared with other ratios. The cofermented maize‐pigeon pea product 60:40 had high amino acid profile than the others.

## 1 INTRODUCTION

Ogi is consumed by adults and children as breakfast meals, and it also serves as a weaning diet (Ashaye, Fasoyiro, & Kehinde, 2000; Amusa et al., 2005). After 5–6 months, breast‐feeding is no longer sufficient to satisfy the nutritional requirements of the growing infant. Beginning from this period, the child needs solid foods to meet increasing nutritional needs (Onofiok & Nnanyelugo, 1998). This period is the weaning period and in Nigeria, ogi (alternatively called pap or akamu) is introduced gradually to the child's diet to supplement nutrition. Fermented maize is very widely utilized as food in African countries and in fact cereals account for as much as 77% of total caloric consumption (Osungbaro, 2009). Maize is rich in carbohydrates and minerals, including potassium and magnesium. It contains trace amounts of lysine and tryptophan, contributing to the low content of protein, and trace amounts of B‐vitamins (USDA, 2012).

Protein deficiency in infants and young children has been shown to have harmful effects on the brain and may have longer term effects on brain function (Omemu & Faniran, 2011). It has been shown to have adverse effects on the immune system, resulting in a higher risk of infections (Bistrian, Blackburn, Scrimshaw, & Flatt, 1975; Omemu, 2011). It also affects gut mucosal function and permeability which, in turn, affects absorption and makes possible bacterial invasion from the gut, which can result in septicemia. Protein deficiency has also been shown to adversely affect kidney function, affecting adversely glomerular and tubular function (Benabe & Martinez‐Maldonado, 1998).

Fortification of food refers to the addition of essential micronutrients to food particularly added to correct specific nutritional deficiencies such as addition of vitamins and iron to breakfast foods (cereals and beverages) and fortification of sugar with vitamin A and fortification of table salt with iodine (Mbaeyi & Onweluzo, 2010). Several studies have shown the enrichment of ogi with different food substances such as bambara groundnut (Mbata, Ikenebomeh, & Alaneme, 2009), pawpaw (Ajanaku et al., 2010), groundnut seed (Ajanaku, Ajanaku, Edobor, & Nwinyi, 2012), soybean (Adeleke & Oyewole, 2010), crayfish (Ajanaku et al., 2013), okra seed meal (Aminigo & Akingbala, 2004), kersting's groundnut (Kerstingiella geocarpah) flour (Aremu, Olaofe, Audu, & Ijalana, 2011), scarlet runner bean (Aremu, Osinfade, Basu, & Ablaku, 2011), cowpea (Ashaye et al., 2000; Oyarekua, 2009), among others. One cheap method of enhancing the nutritive value of ogi is by adding legumes to it.

Legumes are low‐priced sources of protein‐rich foods that have been important in alleviating protein malnutrition and in the tropics; they are the next important food crop after cereals (Ashaye et al., 2000; Oyarekua, 2009). Leguminous seeds include soybean, cowpea, groundnut, pigeon pea (red gram), mucuna (velvet beans), jack bean, oil bean, and chicken pea. The legume and grain families are by far the world's most important sources of food; grains supply starch, whereas legumes which include bean, peas, and alfalfa supply protein and fats. Legumes are rich sources of protein, energy, vitamins, dietary fiber, minerals, and oil, especially the oil‐seeds (Arawande and Borokini, 2010). Cereals are deficient in lysine and tryptophan, but rich in methionine and cysteine, whereas legumes are deficient in sulfur‐containing amino acids such as methionine and cysteine, and rich in lysine (Sodipo & Fashakin, 2011). There is a need to combine the cereals and legumes together so as to complement each other.

Pigeon pea (*Cajanus cajan*) is a locally available, affordable, and underutilized grain legume of the tropics and subtropics (Fasoyiro et al., 2010). Pigeon pea uses include soil improvement, agroforestry, intercropping, food, feed, wood, and pest management (Valenzuela and Smith, 2002). Pigeon pea is reported to contain 20%–22% protein, 1%–2% fat, 65% carbohydrate, and 6.8% ash (Onweluzo & Nwabugwu, 2009) and rich in mineral quality and fiber content. It is known as “*otili*” in the South‐western part of Nigeria. In South‐eastern Nigeria, it is usually consumed in cooked form like cooked beans and most consumers prefer to cook “*fio‐fio*”, as it is usually called, with firewood due to the long period of time it takes to soften. The presence of antinutrients in pigeon pea has limited its utilization. Fermentation process had also been utilized to increase the protein and textural qualities of the seeds (Fasoyiro et al., 2010).

The main objective of this study is to evaluate the nutritional and antinutritional qualities of ogi developed from maize and pigeon pea.

## 2 MATERIALS AND METHODS

### 2.1 Sample collection

The pigeon pea (*Cajanus cajan*) and the dried maize grains (*Zea mays*) used in this study were purchased from Bariga market, Bariga, Lagos State, Nigeria.

### 2.2 Cleaning and weighing of maize/pigeon pea samples

The grains/pea seeds were sorted to separate them from stones, dirt, etc. Six different proportions of maize: Pigeon peas were weighed as follows Table 1.

### 2.3 Fermentation of maize‐pigeon pea blends

**Figure 1:**
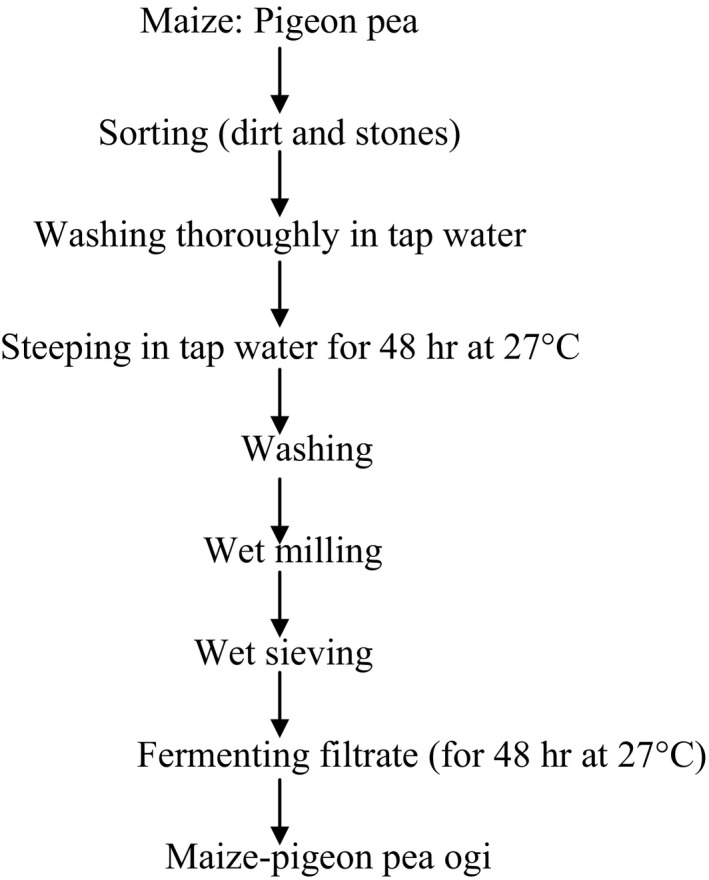
Unit operations for the production of Maize‐pigeon pea ogi

The grains/peas were washed thoroughly and steeped in tap water in the ratio of 1:2 (w/v) in properly labeled plastic buckets with lids. Maize‐pigeon pea ogi was prepared using a modified traditional fermentation method (Odunfa & Adeleye, 1985). The different maize‐pigeon pea blends were steeped for 48 hr, washed, wet‐milled, and then sieved using muslin cloth to separate the pomace from the filtrate. The filtrates were allowed to settle and sour for another 48 hr (Figure 1). During the fermentation (steeping and souring) process, samples were taken at 24 hr interval for microbiological, chemical, nutritional, and antinutritional analysis.

### 2.4 Proximate analysis

Proximate analyses (% crude protein, % fat, % crude fiber, % ash, % moisture content, and % carbohydrates) were determined on samples collected at 24‐hr interval using method described by AOAC (2005).

### 2.5 Nutritional analysis

Samples taken at 24 hr interval throughout the maize‐pigeon pea ogi fermentation process were subjected to mineral and vitamin analysis, and amino acid profiling. The minerals analyzed were potassium, phosphorus, calcium, sodium, magnesium, and iron, whereas the vitamins analyzed were vitamins B_1_, B_2_, and B_3_.

### 2.6 Analysis of minerals

The method of Mbaeyi and Onweluzo (2010) was used in analyzing minerals. Mineral analysis of samples taken from the fermenting maize‐pigeon pea ogi at 24‐hr interval was determined in three phases: sample preparation, sample digestion, and atomic absorption spectrophotometer (AAS) analysis.

#### 2.6.1 Sample preparation

Samples that were in grain form collected during the steeping period of fermentation were washed with distilled water and dried in the oven at 70°C for 3 hr. Afterward, they were blended to get grain powders. Samples in slurry form collected during the souring period of fermentation were weighed directly for the analysis. A solution of HNO_3_ and distilled water H_2_O in the ratio of 3:1 was used to digest the samples in order to free the mineral from their complex forms. Standard serial concentrations of pure forms of the minerals were prepared to standardize the AAS before reading the concentration of minerals. Serial dilutions used were 0.5 mg, 1.0 mg, 2.0 mg, 4.0 mg, and 8.0 mg made from 100 mg/100 ml standard flask.

#### 2.6.2 Sample digestion

Sample (0.67 g) was weighed into a glass beaker and 50 ml of HNO_3_: H_2_O was added to it. The solution was heated in a fume cupboard with Bunsen burner applying gentle heat. The HNO_3_ fumes were allowed to escape gradually until no more fumes were seen. This indicated the end of the digestion. The digested samples were then filtered into a 50 ml standard flask and made up to volume with distilled water and ready for AAS analysis.

#### 2.6.3 Atomic absorption spectrophotometer analysis of digested samples

A standard curve was obtained for each of the minerals using the serially diluted concentration standards using an appropriate lamp particular to a given mineral which was mounted on the AAS. After obtaining the standard curve at a particular wavelength, the digested sample in the container was sucked into the AAS for analysis. At that wavelength which a particular mineral absorbed highest, the molecules were excited and moved to higher energy level. On returning back to their ground state, the excess energy absorbed and given off was observed as the concentration of the minerals. The different minerals analyzed, their lamps and individual wavelengths were potassium (K lamp; 766 nm), phosphorus (P lamp; 213 nm), calcium (Ca lamp; 317 nm), sodium (Na lamp; 589 nm), magnesium (Mg lamp; 279 nm), and iron (Fe lamp; 259 nm).

### 2.7 Analysis of vitamin B_1_ (thiamin)

Vitamin B_1_ was analyzed in samples using the AOAC (2005) method. Accurately weighed 1.5 g of test sample was introduced into a 200 ml volumetric flask; 100 ml of 0.1 N HCL solution was added and the mixture heated in a water bath at 100°C for 30 min. After cooling, the content of the flask was made up to mark with 0.1M HCL solution and mixed thoroughly. The solution was filtered using Whatman No. 1 filter paper. The first 20 ml of the filtrate was discarded. The remaining filtrate (100 ml) was transferred into centrifuge tube containing 0.5 g frankonite powder (a flocculant which precipitate the particles faster during centrifugation) stirred for 10 min using RAM 2718 stirrer, then centrifuged at 5,000 rpm for 5 min to separate layers. The supernatant liquid was discarded while 5 ml of absolute alcohol and 5 ml of the potassium ferric‐cyanide solution in sodium hydroxide solution were added after it was previously frozen at 0°C. A pinkish coloration of mixture was observed after 10 min of mixing, and then 10 ml of toluene solution was added, stirred for 10 min and centrifuged for 10 min at 5,000 rpm. A very clear pink color was transferred to the toluene layer. Thiamine standard (0.5 mg) was prepared and 10 ml of the thiamine standard solution was treated same as sample above. The standard and sample solution was read at 530 nm wavelength using the SP 30UV spectrophotometer (Pye Unican). The amount of thiamine present in each sample was calculated as thus:

$$\begin{array}{cc} \text{Thiamine (mg/100}\;\;\;\text{g)}\;\;= & \frac{\mathrm{absorbance}\mathrm{of}\mathrm{sample}}{\mathrm{absorbance}\mathrm{of}\mathrm{standard}}\times \frac{\mathrm{weight}\mathrm{of}\mathrm{standard}(\mathrm{mg})}{\mathrm{weight}\mathrm{of}\mathrm{sample}(g)}\\ & \times 100 \end{array}$$

### 2.8 Analysis of vitamin B_2_ (riboflavin)

Vitamin B_2_ was analyzed in samples using the AOAC (2005) method. Accurately weighed 1.5 g of sample was introduced into 200 ml volumetric flask; 100 ml of acetic acid: water mixture (50:50) was added and heated on a boiling water bath at 100°C for 30 min. The mixture in the flask was cooled to 20°C, then made up to the mark with acetic acid‐water solution. The mixture was stirred for 10 min using the stirrer and then filtered in the dark. The first 20 ml of the filtrate was discarded, 0.5 mg of riboflavin standard solution was prepared, and 10 ml of the standard solution was transferred into 200 ml volumetric flask and treated similarly as sample above. The fluorescence of the standard and sample solutions was read using spectrophotometer at 460 nm wavelength. The amount of riboflavin in each sample was calculated as follows

$$\begin{array}{cc} \text{Riboflavin (mg/100}\;\;\;\text{g)} & \;=\frac{\text{sample absorbance}}{\text{standard absorbance}}\times \frac{\text{weight of std (mg)}}{\text{weight of sample}}\\ & \times 100 \end{array}$$

### 2.9 Analysis of vitamin B_3_ (niacin)

Vitamin B_3_ was analyzed in samples using the AOAC (2005) method. Sample (1.5 g) was accurately weighed into 200 ml volumetric flask. Hydrochloric acid solution (5 N; 5 ml) was added, and 5.0 ml of dichloromethane and 90 ml of deionized water were added to the mixture, stirred, and heated on a boiling water bath at 100°C for 30 min. It was then cooled and the flask content made up to the mark with distilled water, filtered using Whatman No. 1 filter paper discarding the first 20 ml of the filtrate. The niacin standard solution of 0.5 mg was prepared, and 10 ml of the stock solution was taken and treated same as sample above. The absorbance of the standard and sample solutions were taken at 410 nm wavelength using spectrophotometer and calculation followed thus:

$$\begin{array}{cc} \text{Niacin (mg/100}\;\;\;\text{g)}= & \frac{\text{sample reading}}{\text{standard reading}}\times \frac{\text{standard weight (mg)}}{\text{sample weight}}\\ & \times 100 \end{array}$$

### 2.10 Amino acid profile determination

The amino acid profile of samples taken from the fermenting maize‐pigeon pea ogi at 24 hr interval was determined in four phases: the amino acid extraction phase, the chromatographic phase, the quantitative determination of amino acid profile phase, and the colorimetric analysis of amino acid profile phase.

#### 2.10.1 Extraction of amino acids

Two gram (2 g) samples were weighed using digital chemical balance (model OHAUS precision plus). The samples were blended in a mortar and pestle and transferred into 250 ml beaker. Phosphate buffer solution (0.2 mol/L; 20 ml) pH 7.0 was added. The mixture was stirred for about 3 min and the resulting mixture was centrifuged at 2,000 rpm for 10 min. The supernatant was decanted and poured into separating funnel. The supernatant was shaken three times with 10 ml portion of petroleum ether to remove the organic pigments. The top phase was discarded and the aqueous phase which contained protein and amino acids was retained. Protein was precipitated from the aqueous phase by adding 5 ml of 10% w/v trichloroacetic acid (TCA) to 5 ml extract. The mixture was shaken and kept in the freezer for 10 min. The precipitate formed was removed by centrifugation (2m000*g*) and the filtrate was used for the amino acid profile determination.

#### 2.10.2 Chromatographic analysis of amino acids (TLC technique)

The amino acid content in the extract was separated by thin‐layer chromatography method (TLC technique). Aliquots of 50 μl of the extracts were spotted on Avicel microcrystalline cellulose thin‐layer plates (Whatman analytical plates) along with 20 μl of reference standard mixture. The reference mixture contained lysine, histidine, phenylalanine, methionine, glycine, cysteine, proline, leucine, isoleucine, threonine, tyrosine, valine, arginine, tryptophan, and glutamic acid (BDH and Sigma chemicals), each present at a concentration of 0.1% (w/v). One‐dimensional ascending chromatography was done, and the solvent system employed for the separation was n‐butanol‐glacial acetic acid‐water at a ratio of 4:1:2 (v/v). After 4 hr of separation the chromatograms were air‐dried and the amino acids were located by spraying with locating reagent, 0.2% (w/v) of Ninhydrin in ethanol. The sprayed chromatograms were allowed to air‐dry and later oven‐dried at 100°C for 5 min for the spots to be clearly identified. The separated amino acids were identified using the reference standard spotted alongside (Mikes and Chalmers, 1981).

#### 2.10.3 Quantitative determination of amino acid profile

The quantitative determination of the amino acids (profile) was done through colorimetric method of Rosen (1957) as reported by Opere et al., (2012). The estimation of the amino acids was by use of the guide strip technique where developed thin‐layer chromatography plates were used in locating the positions of amino acids in unsprayed plates. The squares containing amino acids were cut‐out and eluted with 5 ml distilled water at 70°C for 2 hr; the cellulose powder was removed by centrifugation at 5,000 rpm for 5 min. The supernatants were decanted and kept for the colorimetric analysis of amino acid profiles.

#### 2.10.4 Colorimetric analysis of amino acid profile

The extracts obtained above from the samples were used for amino acid profiles analysis using the modified ninhydrin colorimetric analysis method of Rosen (1957) as reported by Opere et al., (2012). To 1 ml of the diluted extracts of each amino acid was added 0.5 ml cyanide‐acetate buffer (pH 5.4) and 0.5 ml 3% (w/v) Ninhydrin in methylcellosolve. The mixture was heated in a boiling water bath at 100°C for 15 min. Immediately after the mixture was removed from the water bath, 5.0 ml iso‐propyl alcohol‐water mixture (ratio 1:1) was added as diluents and mixed by shaking vigorously, then cooled to room temperature (25°C). The amino acid profile was estimated by determining the optical density at 570 nm wavelength using UV/visible spectrophotometer. The blank was similarly treated as sample above and used as the control to set the absorbance to zero (distilled water). The amount of amino acid content was each calculated from the standard curve of known concentration of leucine (10 mg/ml).

### 2.11 Antinutritional analysis

Samples taken at 24‐hr interval throughout the maize‐pigeon pea ogi fermentation process were subjected to antinutritional analysis. The antinutrients analyzed were phytate, tannins, and trypsin inhibitors.

#### 2.11.1 Analysis of phytate

Phytate determination in samples followed the method AOAC (2003). Phytate was extracted with 0.5 mol/L (HNO_3_) solution and digested with 0.5 ml of (HClO_4_) perchloric acid. The digested sample was made up to 25 ml volume with distilled water in a standard volumetric flask. From this extract, 2.5 ml of sample was taken and added to 2.5 ml of nitric acid, and the phytic phosphorus present in the extract reacted with 2.5 ml of vanadium molybdate (solution) reagent to produce a yellow‐orange complex. The absorbance (OD) was measured at 460 nm wavelength using a spectrophotometer (P7 UV/Vis spectrophotometer). The phytate content was then calculated from the 2 mg of phytic acid standard concentration with a reagent blank treated as sample above.

$$\begin{array}{cc} \text{Phytate (mg/100}\;\;\;\text{g)}= & \frac{\text{sample absorbance}\times \text{standard concentration}}{\text{standard absorbance}\times \text{weight of sample}}\\ & \times 100 \end{array}$$

#### 2.11.2 Analysis of tannin

Tannin content was determined by the AOAC (2005) method. Sample (5 g) was dispensed in 50 ml of distilled water and shaken. The mixture was allowed to stand for 30 min at 28°C before it was filtered through Whatman no.4 grade of filter paper. The extract (2 ml) was dispensed into a 50 ml volumetric flask. Similarly, 2 ml standard tannic solution (0.1 mg/ml tannic acid) and 2 ml distilled water were put in a separate volumetric flask to serve as standard. 2.5 ml of saturated sodium carbonate (Na_2_CO_3_) solution and 1 ml of Folin‐C reagent were added to each flask and volume made up to 50 ml and mixed well. After standing for 1½ hr, the sample was filtered using Whatman no.4 grade of filter paper and the absorbance measured at 760 nm against reagent blank.

$$\text{Tannin (mg/100}\;\;\;g)=\frac{\text{standard concentration}\times \text{sample absorbance}}{\text{standard absorbance}\times \text{weight of sample}}$$

#### 2.11.3 Analysis of trypsin inhibitor activity (TIA)

Trypsin inhibitor activity (TIA) of samples taken from the fermenting maize‐pigeon pea ogi at 24 hr interval was analyzed in two phases: the crude inhibitor (trypsin) was extracted from jatropha leaves before the assay of TIA was carried out.

##### Extraction of crude inhibitor

Jatropha leaves (50 g) were weighed and cut into pieces. The leaves were homogenized in a blender containing 50 ml of sodium dithionite (0.7% w/v). The homogenate was left to stand for about 1 hr and then filtered through layers of cheese cloth into a beaker and thereafter centrifuged at 1,000 rpm for 5 min to remove fine particles. The resulting supernatant was stored in the refrigerator.

##### Assay of TIA

Trypsin inhibition activity was assayed by the procedure of AOAC (2005). Sample extract (0.1 ml) and 0.9 ml of 0.1 mol/L phosphate buffer pH 8.0 was mixed with the same volume of trypsin solution and preincubated at 37°C for 5 min; 1 ml of 0.03% (w/v) Bovine Serum Albumin (BSA) was added to the mixture and incubated for 30 min at 37°C after which the reaction was stopped by the addition of 2 ml of 5% (w/v) Trichloroacetic acid (TCA) solution. The mixture was filtered. To 1 ml of the filtrate, 5 ml of 0.55 mol/L Na_2_CO_3_ and 0.1 ml of Folin‐C reagent. The resulting color absorbance was determined at 660 nm wavelength. Standard sample was prepared in the absence of inhibitor.

$$\%\text{Trypsin inhibition activity}=\frac{T-T^{\circ }}{T}\times 100$$

where, *T*, absorbance in the absence of inhibitor; *T*°, absorbance in the presence of inhibitor.

### 2.12 Statistical analysis

Data obtained were subjected to analysis of variance (ANOVA) at α = 0.05 level of significance with the use of the Statistical Package for Social Sciences (SPSS) version 16.0. Significant means (*p *<* *.05) were separated using Duncan multiple range test. Graphs and charts were plotted with the use of Microsoft excel 2007 software.

## 3 RESULTS

### 3.1 Proximate determination

Based on the result of the sensory evaluation of the different fermenting maize‐pigeon pea ogi blends, the proximate, mineral, vitamin, antinutritional, and amino acid profile analysis were carried out on 60:40 and 70:30 maize‐pigeon pea ogi blends with 100:0 serving as control (Table 1).

**Table 1:** Materials used for the production of weaning food

Sample ratio	Maize (g)	Pigeon pea (g)
100:0	1,000	0
90:10	900	100
80:20	800	200
70:30	700	300
60:40	600	400
50:50	500	500

### 3.2 Changes in moisture, fat, and ash contents

Changes in moisture, fat, and ash contents of the different fermenting maize‐pigeon pea ogi blends are shown in Table 2. At the 0‐hr steeping period, the moisture content of 100:0 maize‐pigeon pea ogi (1.04%) was the highest and was significantly (*p *≤* *.05) different from the others. As fermentation progressed, the moisture contents significantly (*p *≤* *.05) decreased. At 24 hr souring, there was a significant (*p *≤* *.05) decrease in moisture content of the different maize‐pigeon pea ogi blends and this decrease continued till the end of fermentation (48 hr souring). At the end of fermentation, 100:0 maize‐pigeon pea ogi had the lowest moisture content (0.75%) and 60:40 maize‐pigeon pea ogi had the highest moisture content (1.98%).

**Table 2:** Changes in moisture, fat, and ash contents of the different fermenting maize‐pigeon pea ogi blends

Time (h)		Moisture content (%)			Fat content (mg/100 g)			Ash content (mg/100 g)		
		100:0	70:30	60:40	100:0	70:30	60:40	100:0	70:30	60:40
Steeping phase	0	1.04 ± 0.01^a^	1.00 **± **0.00^b^	1.00 ± 0.00^b^	7.60 ± 0.01^c^	9.11 ± 0.02^b^	13.36 ± 0.01^a^	0.66 ± 0.00^c^	0.72 ± 0.02^b^	0.77 ± 0.00^a^
	24	1.30 ± 0.00^c^	1.60 ± 0.01^b^	1.80 ± 0.01^a^	9.11 ± 0.01^c^	13.99 ± 0.01^b^	15.14 ± 0.00^a^	0.85 ± 0.01^c^	0.90 ± 0.00^b^	1.09 ± 0.01^a^
	48	2.90 ± 0.00^c^	3.20 ± 0.01^b^	3.60 ± 0.01^a^	9.21 ± 0.02^c^	15.09 ± 0.01^b^	18.37 ± 0.01^a^	0.94 ± 0.01^c^	1.76 ± 0.00^b^	1.97 ± 0.01^a^
Souring phase	0	3.54 ± 0.01^c^	3.81 ± 0.01^b^	4.00 ± 0.01^a^	10.11 ± 0.01^c^	15.16 ± 0.00^b^	18.39 ± 0.00^a^	0.92 ± 0.01^c^	1.70 ± 0.02^b^	1.95 ± 0.01^a^
	24	2.90 ± 0.01^c^	3.05 ± 0.00^b^	3.15 ± 0.01^a^	10.15 ± 0.01^c^	15.27 ± 0.01^b^	19.05 ± 0.01^a^	1.04 ± 0.02^c^	1.98 ± 0.01^b^	2.56 ± 0.01^a^
	48	0.75 ± 0.00^c^	1.41 ± 0.00^b^	1.98 ± 0.00^a^	10.15 ± 0.01^c^	16.51 ± 0.01^b^	19.27 ± 0.01^a^	1.07 ± 0.01^c^	2.55 ± 0.01^b^	2.98 ± 0.00^a^

Values are mean ± standard deviation of triplicate determinations. Means on the same row with different sets of superscripts are statistically different (*p* ≤ .05).

The low moisture content of the different fermented products (100:0%–0.75%; 70:30%–1.41%; 60:40%–1.98%) at the end of the 96 hr fermentation in this study indicate that they would have good keeping quality because food spoiling microflora thrives where there is adequate moisture (Eno‐Obong & Carnovale, 1992). The reduced moisture content is in line with report of Otunola, Sunny‐Roberts, and Solademi (2007). The recommended dietary allowance (RDA) for moisture in foods is ≤5% (FAO/WHO, 1998) and all the fermented products, including the fortified products, had moisture contents within this range.

Changes in the fat content of the different fermenting maize‐pigeon pea ogi blends were significantly different at the different stages of fermentation. At the 0 hr steeping period, the 60:40 maize‐pigeon pea ogi (13.36 mg/100 g) had the highest fat content; followed by the 70:30 maize‐pigeon pea ogi (9.11 mg/100 g), whereas 100:0 maize‐pigeon pea ogi (7.60 mg/100 g) had the least fat content. As fermentation progressed, the fat content significantly (*p *≤* *.05) increased in all the maize‐pigeon pea ogi blends.

There was increase in fat content observed during fermentation in this study. This is in line with Otunola et al. (2007) that showed increases in fat content of maize‐ogi fortified with okra seed flour. Increased fat content was also reported by Mbata et al. (2009). The improvement to the fat content of the fortified ogi products may be due to increase in activity of lipolytic enzymes in the fermentation medium which hydrolyzes fat to glycerol and fatty acids (Modu et al., 2013). The free fatty acids are used by the fermenting organisms for the synthesis of new lipids (Ezeokonkwo, 2004). The fat requirement for neonates is essential for the development of brain especially for children under 2 years of age (Ajanaku et al., 2013). The recommended dietary allowance (RDA) for fat in foods is ≥2.0 mg/100 g (FAO/WHO, 1998) and only the fortified products had fat contents (70:30–16.51 mg/100 g; 60:40–19.27 mg/100 g) within this range.

At the end of fermentation (48 hr souring), 60:40 maize‐pigeon pea ogi (19.27 mg/100 g) had the highest fat content; followed by 70:30 maize‐pigeon pea ogi (16.51 mg/100 g), whereas 100:0 maize‐pigeon pea ogi (10.15 mg/100 g) had the least fat content.

Changes in the ash content of the different fermenting maize‐pigeon pea ogi blends were significantly different at the different stages of fermentation. At the 0 hr steeping period, 60:40 maize‐pigeon pea ogi (0.77 mg/100 g) had the highest ash content; followed by 70:30 maize‐pigeon pea ogi (0.72 mg/100 g) and 100:0 maize‐pigeon pea ogi (0.66 mg/100 g) had the least ash content. As fermentation progressed, the ash content significantly (*p *≤* *.05) increased in all maize‐pigeon pea ogi blends, although there was a slight decrease at the beginning of souring. At the end of fermentation (48 hr souring), 60:40 maize‐pigeon pea ogi (1.95 mg/100 g) had the highest ash content; followed by 70:30 maize‐pigeon pea ogi (1.70 mg/100 g), and 100:0 maize‐pigeon pea ogi (0.92 mg/100 g) had the least ash content.

### 3.3 Changes in crude fiber, crude protein, and carbohydrate contents

Changes in crude fiber, crude protein, and carbohydrate contents of the different fermenting maize‐pigeon pea ogi blends are shown in Table 3.

**Table 3:** Changes in crude fiber, crude protein, and carbohydrate contents of the different fermenting maize‐pigeon pea ogi blends

Time (h)		Crude fiber content (mg/100 g)			Crude protein content (mg/100 g)			Carbohydrate content (mg/100 g)		
		100:0	70:30	60:40	100:0	70:30	60:40	100:0	70:30	60:40
Steeping phase	0	0.22 ± 0.01^c^	0.39 ± 0.00^b^	0.69 ± 0.00^a^	10.32 ± 0.01^c^	14.61 ± 0.00^b^	16.14 ± 0.00^a^	80.16 ± 0.01^a^	74.17 ± 0.01^b^	68.04 ± 0.01^c^
	24	0.29 ± 0.01^c^	0.45 ± 0.02^b^	0.72 ± 0.01^a^	10.65 ± 0.01^c^	16.95 ± 0.00^b^	19.11 ± 0.01^a^	77.80 ± 0.02^a^	66.11 ± 0.01^b^	62.14 ± 0.01^c^
	48	0.31 ± 0.01^c^	0.49 ± 0.00^b^	0.79 ± 0.01^a^	12.43 ± 0.01^c^	29.82 ± 0.01^b^	32.69 ± 0.01^a^	74.21 ± 0.01^a^	42.58 ± 0.01^b^	42.58 ± 0.01^b^
Souring phase	0	0.21 ± 0.00^c^	0.47 ± 0.01^b^	0.75 ± 0.00^a^	12.00 ± 0.02^c^	25.23 ± 0.00^b^	30.65 ± 0.00^a^	73.22 ± 0.01^a^	53.63 ± 0.02^b^	44.26 ± 0.01^c^
	24	0.22 ± 0.01^c^	0.48 ± 0.00^b^	0.74 ± 0.01^a^	13.92 ± 0.00^c^	22.95 ± 0.00^b^	25.87 ± 0.01^a^	71.77 ± 0.01^a^	56.27 ± 0.06^b^	48.63 ± 0.01^c^
	48	0.25 ± 0.00^c^	0.50 ± 0.01^b^	0.73 ± 0.01^a^	14.95 ± 0.00^c^	21.14 ± 0.01^b^	22.79 ± 0.01^a^	72.83 ± 0.01^a^	57.89 ± 0.01^b^	53.25 ± 0.01^c^

Values are mean ± standard deviation of triplicate determinations. Means on the same row with different sets of superscripts are statistically different (*p* ≤ .05).

Supplementation of ogi with pigeon pea was shown to have a significant effect (*p *≤* *.05) on the crude fiber content of the products from the beginning (0 hr steeping) to the end (48 hr souring) of fermentation. At the 0 hr steeping period, the crude fiber content of 60:40 maize‐pigeon pea ogi (0.69 mg/100 g) was the highest and was significantly different (*p *≤* *.05) from that of 70:30 maize‐pigeon pea ogi (0.39 mg/100 g), whereas 100:0 maize‐pigeon pea ogi (0.22 mg/100 g) had the least crude fiber content. The crude fiber content increased in all three maize‐pigeon pea ogi.

The finding of increase in ash in the fermented ogi products in this study is in contrast to the report of Modu et al., 2013. Ajanaku et al., 2013 and Mbata et al., 2009, reported increases in ash content in their fortified products. Ash content of a food is a determinant of the mineral content of that particular food. The higher the ash content, the more the mineral content of the food (Ukegbu & Anyika, 2012). The recommended dietary allowance (RDA) for ash in foods is ≤5.0 mg/100 g (FAO/WHO, 1998) and all the fermented products had ash contents (100:0–1.07 mg/100 g; 70:30–2.55 mg/100 g; 60:40–2.98 mg/100 g) within this range.

Dietary fiber consists primarily of the indigestible complex carbohydrate of cell wall in plants. High dietary fiber can have some beneficial biological effects such as laxative effect on GIT, increased fecal bulk and help reduce plasma cholesterol level (Okoye, 1992). In this study, crude fiber values were found to be higher in the fortified ogi products (70:30–0.50 mg/100 g; 60:40–0.73 mg/100 g) than in the unfortified ogi (100:0–0.25 mg/100 g). The recommended dietary allowance (RDA) for crude fiber in foods for infants (6–12 months) is 4.0 mg/100 g (FAO/WHO, 1998), implying that the crude fiber contents of the fermented products in this study did not meet the recommended allowance for infants.

The recommended dietary allowance (RDA) for crude protein in foods is ≥16.0 mg/100 g (FAO/WHO, 1998) and the fermented products in this study (70:30–21.14 mg/100 g; 60:40–22.79 mg/100 g) had crude protein contents within this range, except the unfortified product (100:0–14.95 mg/100 g) which was below the recommended range. It can be seen that the protein content of the pigeon pea fortified ogi samples were significantly higher than that of the unfortified sample. This could be as a result of improvement in the protein content of the maize during fermentation by the addition of pigeon pea. This is similar to the reports of Ijarotimi & Aroge, 2005; Modu et al., 2013. Reports have also shown that protein quality is synergististically improved in cereal‐legume blends due to the contribution of lysine by legume and methionine by cereal (Wakil & Kazeem, 2012).

The decrease in carbohydrate content from the beginning to the end of fermentation observed in this study agreed with the observation that addition of legume decreases the carbohydrate content of maize‐based traditional foods (Mbata et al., 2009; Sefa‐Dedeh, Sakyi‐Dawson, Afoakwa, Andoh‐Kumi, & Tano‐Debrah, 2001). The recommended dietary allowance (RDA) for carbohydrate in foods is ≥60 mg/100 g (FAO/WHO, 1998) and the fortified products (60:40–53.25 mg/100 g; 70:30–57.89 mg/100 g) met only 88.75%–96.48% of this requirement.

### 3.4 Determination of minerals

#### 3.4.1 Changes in potassium (K), phosphorus (P), and calcium (Ca) contents

Changes in potassium, phosphorus, and calcium contents of the different fermenting maize‐pigeon pea ogi blends are shown in Table 4. The result showed that there was significant effect (*p *≤* *.05) of fortification on the potassium, phosphorus, and calcium contents of the different fermenting maize‐pigeon pea ogi blends.

**Table 4:** Changes in potassium (K), phosphorus (P), and calcium (Ca) contents of the different fermenting maize‐pigeon pea ogi blends

Time (h)		K (mg/100 g)			P (mg/100 g)			Ca (mg/100 g)		
		100:0	70:30	60:40	100:0	70:30	60:40	100:0	70:30	60:40
Steeping phase	0	274.70 ± 0.10^c^	287.01 ± 0.03^b^	298.50 ± 0.02^a^	249.86 ± 0.04^c^	261.45 ± 0.05^b^	272.95 ± 0.05^a^	73.55 ± 0.05^c^	155.05 ± 0.05^b^	174.45 ± 0.05^a^
	24	300.32 ± 0.03^c^	312.35 ± 0.05^b^	323.88 ± 0.01^a^	275.39 ± 0.01^c^	287.39 ± 0.01^b^	298.85 ± 0.05^a^	127.08 ± 0.02^c^	218.79 ± 0.02^b^	249.73 ± 0.21^a^
	48	312.76 ± 0.04^c^	323.00 ± 0.00^b^	334.33 ± 0.03^a^	287.85 ± 0.05^c^	297.60 ± 0.00^b^	309.40 ± 0.00^a^	178.00 ± 0.00^c^	327.14 ± 0.01^b^	367.88 ± 0.02^a^
Souring phase	0	310.00 ± 0.05^c^	319.45 ± 0.05^b^	334.00 ± 0.00^a^	287.15 ± 0.05^c^	295.12 ± 0.03^b^	307.87 ± 0.03^a^	105.37 ± 0.02^c^	247.44 ± 0.01^b^	277.85 ± 0.05^a^
	24	285.00 ± 0.00^c^	292.15 ± 0.05^b^	302.06 ± 0.02^a^	262.50 ± 0.00^c^	281.10 ± 0.10^b^	286.02 ± 0.03^a^	167.38 ± 0.03^c^	292.50 ± 0.00^b^	317.99 ± 0.01^a^
	48	277.33 ± 0.01^c^	283.50 ± 0.01^b^	289.52 ± 0.03^a^	253.35 ± 0.05^c^	271.70 ± 0.00^b^	272.96 ± 0.01^a^	199.61 ± 0.01^c^	329.92 ± 0.03^b^	344.83 ± 0.01^a^

Values are mean ± standard deviation of triplicate determinations. Means on the same row with different sets of superscripts are statistically different (*p* ≤ .05).

At the beginning of fermentation (0 hr steeping period), the potassium content of the different fermenting maize‐pigeon pea ogi blends ranged from 274.70 to 298.50 mg/100 g, with 100:0 maize‐pigeon pea ogi having the lowest potassium content (274.70 mg/100 g) and 60:40 maize‐pigeon pea ogi having the highest (298.50 mg/100 g). As fermentation progressed, the potassium content increased in all the different fermenting maize‐pigeon pea ogi blends, with a range of 312.76–334.32 mg/100 g at the 48 hr steeping period, until the beginning of souring (0 hr souring) when it began to drop. At the end of fermentation (48 hr souring), the potassium content had dropped to 277.33 (100:0 maize‐pigeon pea ogi)—289.52 (60:40 maize‐pigeon pea ogi) mg/100 g.

From this study, it can be observed that addition of pigeon pea to maize in the production of ogi yields fortified products with improved valuable mineral content. This is also substantiated by the increased ash content of the fortified maize—pigeon pea ogi products. Some researchers (Adeyemi & Soluade, 1993; Enujiugha, 2006) also observed significant increase in ash and mineral contents in African oil bean–ogi and pawpaw–ogi, respectively.

Potassium is an essential nutrient needed for maintenance of total body fluid volume, acid and electrolyte balance, and normal cell function (Young, 2001). Low potassium intake has been associated with a lot of noncommunicable diseases including hypertension, cardiovascular diseases, chronic kidney stone formation, and low bone‐mineral density in children (WHO, 2003; WHO, 2012). As stated in Ukegbu and Anyika (2012), the recommended dietary allowance of potassium for infants less than 1 year is 500 mg/100 g. All the fermented products in this study did not meet this requirement. The fortified diet 60:40 (289.52 mg/100 g) met only 57.9% of the RDA for potassium, whereas 70:30 (283.50 mg/100 g) met only 56.7%.

Phosphorus is fundamental to growth, maintenance, and repair of all body tissues, and is necessary, along with calcium and magnesium, for proper growth and formation of bones in infants and children (Heaney and Nordin, 2002). It also functions to buffer body fluids to maintain a normal pH, to temporarily store and transfer energy derived from metabolic fuels, and to activate many catalytic proteins through phosphorylation (Dickinson, 2004). The recommended dietary allowance (RDA) for phosphorus in infants’ food is ≥180 mg/100 g (FAO/WHO, 1998; Haque, Rulquin, & Lemosquet, 2013) and all the fermented products had phosphorus contents within this range.

According to FAO/WHO (1998) as reported in Oyarekua (2011b), the recommended dietary allowance (RDA) of calcium in infants’ food is 295 mg/100 g, and all the different fermented products met the RDA except the unfortified diet 100:0 (199.61 mg/100 g). Calcium in conjunction with phosphorus, magnesium, vitamin A, C, and D, chlorine, and protein are all involved in bone and teeth formation (Heaney RP, Recker RR, Watson P, Lappe JM.). It is also important in blood clotting, muscle contraction, and in certain enzymes in metabolic processes (Adeyeye & Fagbohun, 2005). Its deficiency can lead to rickets in children (Oyarekua, 2011b), a reduction in bone mass and the development of osteoporosis (Dickinson, 2004).

#### 3.4.2 Changes in sodium (Na), magnesium (Mg), and iron (Fe) contents

Changes in sodium, magnesium, and iron contents of the different fermenting maize‐pigeon pea ogi blends are shown in Table 5. A one‐way analysis of variance revealed that there was significant effect (*p *≤* *.05) of fortification on the sodium, magnesium, and iron contents of the different fermenting maize‐pigeon pea ogi blends.

**Table 5:** Changes in sodium (Na), magnesium (Mg), and iron (Fe) contents of the different fermenting maize‐pigeon pea ogi blends

Time (h)		Na (mg/100 g)			Mg (mg/100 g)			Fe (mg/100 g)		
		100:0	70:30	60:40	100:0	70:30	60:40	100:0	70:30	60:40
Steeping phase	0	54.93 ± 0.03^c^	66.28 ± 0.13^b^	77.91 ± 0.01^a^	77.55 ± 0.05^c^	189.02 ± 0.02^b^	200.51 ± 0.01^a^	3.68 ± 0.02^c^	5.21 ± 0.01^b^	6.72 ± 0.02^a^
	24	79.43 ± 0.02^c^	90.35 ± 0.05^b^	101.58 ± 0.02^a^	143.03 ± 0.03^c^	256.53 ± 0.03^b^	296.15 ± 0.06^a^	15.21 ± 0.01^c^	17.93 ± 0.08^b^	21.35 ± 0.00^a^
	48	91.85 ± 0.05^c^	96.88 ± 0.03^b^	109.45 ± 0.05^a^	178.45 ± 0.05^c^	390.95 ± 0.05^b^	425.35 ± 0.05^a^	20.89 ± 0.01^c^	22.87 ± 0.06^b^	27.80 ± 0.00^a^
Souring phase	0	90.97 ± 0.02^c^	94.99 ± 0.01^b^	103.41 ± 0.00^a^	133.55 ± 0.05^c^	301.99 ± 0.01^b^	334.20 ± 0.01^a^	19.03 ± 0.03^c^	20.23 ± 0.01^b^	27.85 ± 0.05^a^
	24	72.98 ± 0.02^c^	85.85 ± 0.00^b^	89.15 ± 0.05^a^	170.89 ± 0.01^c^	329.99 ± 0.00^b^	357.22 ± 0.01^a^	17.05 ± 0.05^a^	15.15 ± 0.00^b^	14.50 ± 0.00^c^
	48	65.97 ± 0.00^c^	78.64 ± 0.01^b^	81.72 ± 0.07^a^	189.68 ± 0.02^c^	355.82 ± 0.03^b^	373.89 ± 0.01^a^	15.90 ± 0.05^a^	14.24 ± 0.04^b^	14.05 ± 0.00^c^

Values are mean ± standard deviation of triplicate determinations. Means on the same row with different sets of superscripts are statistically different (*p* ≤ .05).

At the 0 hr steeping period, the sodium content of the different fermenting maize‐pigeon pea ogi blends ranged from 54.93 to 77.91 mg/100 g, with 100:0 maize‐pigeon pea ogi having the lowest sodium content and 60:40 maize‐pigeon pea ogi having the highest. As fermentation progressed, the sodium content increased in all set‐ups, with a range of 91.85–109.45 mg/100 g at the 48 hr steeping period, until the beginning of souring (0 hr souring) when it began to drop. At the end of fermentation (48 hr souring), the sodium content had dropped to 65.97 (100:0 maize‐pigeon pea ogi)– 81.72 (60:40 maize‐pigeon pea ogi) mg/100 g.

The magnesium content of the different fermenting maize‐pigeon pea ogi blends at the beginning of fermentation (0 hr steeping) ranged from 77.55 to 200.51 mg/100 g, with 100:0 maize‐pigeon pea ogi having the lowest magnesium content and 60:40 maize‐pigeon pea ogi having the highest. As fermentation progressed, the magnesium content increased in all set‐ups, with a range of 178.45–425.35 mg/100 g at 48 hr steeping. It was observed that at the beginning of souring (0 hr souring), the magnesium content in all fermentation set‐ups dropped slightly, but increased afterward. At the end of fermentation (48 hr souring), the magnesium content ranged from 189.68 mg/100 g in 100:0 maize‐pigeon pea ogi to 373.89 mg/100 g in 60:40 maize‐pigeon pea ogi.

As stated in Ukegbu and Anyika (2012), the recommended dietary allowance of sodium for infants less than 1 year is 120 mg/100 g. The fortified product 60:40 (81.72 mg/100 g) met only 68.1% of the RDA for sodium, whereas 70:30 (78.64 mg/100 g) met 65.5%. Sodium, together with chloride functions in maintenance of extracellular fluids (keeping the water and electrolyte balance of the body) and blood pressure, and also required for nerve and muscle functioning (Oyarekua, 2011b).

The magnesium content of the different fermentation set‐ups was observed to increase significantly throughout the period of fermentation, with 189.68 mg/100 g for product 100:0; 355.82 mg/100 g for 70:30; and 373.89 mg/100 g for 60:40, at the end of fermentation. An increased level of magnesium was also reported in Aremu, Olaofe, et al. (2011) and Aremu, Osinfade, et al. (2011). The recommended dietary allowance (RDA) of magnesium for infants is 350 mg/100 g (Oyarekua, 2011b), and all the fermented products met the RDA except the unfortified product 100:0. Magnesium is the most abundant ion in plant cells. It is needed for many different enzyme systems in the body. It is also important in the formation of adenosine triphosphate (ATP), storage of carbohydrates, fats, and protein, and also needed in nerve and muscle activity, as it maintains electrical potential in nerves (Oyarekua, 2011b).

At the beginning of fermentation (0 hr steeping period), the iron content of the different fermenting maize‐pigeon pea ogi blends ranged from 3.68 to 6.72 mg/100 g, with 100:0 maize‐pigeon pea ogi having the lowest iron content and 60:40 maize‐pigeon pea ogi having the highest. As fermentation progressed, the iron content increased in all set‐ups, with a range of 20.89–27.80 mg/100 g at the 48 hr steeping period. It was observed that at the beginning of souring (0 hr souring), the iron content in most of the fermentation set‐ups dropped slightly (100:0 and 70:30 maize‐pigeon pea ogi blends), but increased afterward. At the 48 hr souring period which marked the end of the fermentation process, the iron content ranged from 14.05 mg/100 g in 60:40 maize‐pigeon pea ogi to 15.90 mg/100 g in 100:0 maize‐pigeon pea ogi.

An increased level of iron at the end of the fermentation period, observed in this study, was also reported in Aremu, Olaofe, et al. (2011) and Aremu, Osinfade, et al. (2011). The recommended dietary allowance (RDA) for iron in infants’ foods is ≥10.0 mg/100 g (Adeyeye & Faleye, 2004; FAO/WHO, 1998) and all the fermented products had iron contents within this range. Iron is a component of hemoglobin that transports oxygen to body tissues and is needed for red blood cell synthesis, respiration, and growth. Its deficiency can lead to anemic conditions in children (Dickinson, 2002).

### 3.5 Determination of vitamin B_1_, B_2_, and B_3_ contents

Changes in vitamin B_1_, B_2_, and B_3_ contents of the different fermenting maize‐pigeon pea ogi blends are presented in Table 6. The result showed that there was significant effect (*p *≤* *.05) of fortification on the vitamin B_1_, B_2_, and B_3_ contents of the different fermenting maize‐pigeon pea ogi blends.

**Table 6:** Changes in vitamin B_1_, B_2_, and B_3_ contents of the different fermenting maize‐pigeon pea ogi blends

Time (h)		Vitamin B_1_ (mg/100 g)			Vitamin B_2_ (mg/100 g)			Vitamin B_3_ (mg/100 g)		
		100:0	70:30	60:40	100:0	70:30	60:40	100:0	70:30	60:40
Steeping phase	0	0.32 ± 0.01^c^	0.64 ± 0.00^b^	0.75 ± 0.01^a^	0.14 ± 0.00^c^	0.69 ± 0.00^b^	0.87 ± 0.00^a^	1.94 ± 0.03^c^	3.19 ± 0.03^b^	3.54 ± 0.03^a^
	24	0.36 ± 0.01^c^	0.71 ± 0.01^b^	0.81 ± 0.00^a^	0.20 ± 0.01^c^	0.85 ± 0.00^b^	0.99 ± 0.01^a^	1.86 ± 0.00^c^	5.26 ± 0.00^b^	6.86 ± 0.05^a^
	48	0.38 ± 0.01^c^	0.81 ± 0.01^b^	0.98 ± 0.01^a^	0.26 ± 0.01^c^	1.30 ± 0.00^b^	1.51 ± 0.01^a^	2.06 ± 0.00^c^	6.46 ± 0.00^b^	7.79 ± 0.03^a^
Souring phase	0	0.43 ± 0.01^c^	0.60 ± 0.00^b^	0.77 ± 0.00^a^	0.27 ± 0.01^c^	0.71 ± 0.00^b^	0.95 ± 0.01^a^	2.11 ± 0.00^c^	2.41 ± 0.00^b^	2.74 ± 0.03^a^
	24	0.51 ± 0.01^c^	1.04 ± 0.01^b^	1.18 ± 0.01^a^	0.33 ± 0.01^c^	1.25 ± 0.01^b^	1.32 ± 0.01^a^	2.16 ± 0.00^c^	4.11 ± 0.00^b^	5.91 ± 0.00^a^
	48	0.57 ± 0.01^c^	1.27 ± 0.00^b^	1.34 ± 0.02^a^	0.37 ± 0.00^c^	1.33 ± 0.00^b^	1.40 ± 0.00^a^	2.21 ± 0.00^c^	4.66 ± 0.00^b^	6.89 ± 0.03^a^

Values are mean ± standard deviation of triplicate determinations. Means on the same row with different sets of superscripts are statistically different (*p* ≤ .05).

The 60:40 maize‐pigeon pea ogi had the highest vitamin B_1_ content (0.75 mg/100 g) at the beginning of fermentation (0 hr steeping); followed by 70:30 maize‐pigeon pea ogi (0.64 mg/100 g) and 100:0 maize‐pigeon pea ogi had the least vitamin B_1_ content (0.32 mg/100 g). There was an evident increase in the vitamin B_1_ content as fermentation progressed. A slight decrease was noticeable at the beginning of souring but values increased as souring progressed.

This study determined the vitamin B content of maize ogi and the fortified maize‐pigeon pea ogi products as according to USDA (2012), maize contains trace amounts of vitamins, especially the B‐vitamins. The fortified products at the end of fermentation were observed to have higher vitamin B_1_ content (70:30–1.27 mg/100 g; 60:40–1.34 mg/100 g) than the control (100:0–0.57 mg/100 g). According to Richardson (1997), the recommended dietary allowance (RDA) of vitamin B_1_ for infants is 1.2 mg/100 g and only the fortified products met this requirement. Vitamin B_1_ (thiamin) is a part of the coenzyme, thiamin pyrophosphate (TPP) that plays critical role in carbohydrate metabolism (the breakdown of glucose for energy). It also acts as coenzyme in the metabolism of the amino acids leucine, isoleucine, and valine. Deficiency in thiamin results in beriberi (Institute of Medicine (US) Standing Committee on the Scientific Evaluation of Dietary Reference Intakes, 1998). The fortified products at the end of fermentation were observed to have higher vitamin B_2_ content (70:30–1.33 mg/100 g; 60:40–1.40 mg/100 g) than the control (100:0–0.37 mg/100 g). According to Richardson (1997), the recommended dietary allowance (RDA) of vitamin B_2_ for infants is 1.3 mg/100 g. Again, only the fortified products met this requirement. Vitamin B_2_ (riboflavin) is a part of the coenzymes, flavin mononucleotide (FMN), and flavin adenine dinucleotide (FAD), needed for oxidation/reduction reactions, including those involved in energy production. Deficiency results in cracks and sores around the mouth and nose, sore throat, magenta tongue, and skin rash, among others (Institute of Medicine (US) Standing Committee on the Scientific Evaluation of Dietary Reference Intakes, 1998). The fortified products at the end of fermentation were also observed to have higher vitamin B_3_ content (70:30–4.66 mg/100 g; 60:40–6.89 mg/100 g) than the control (100:0–2.21 mg/100 g). According to Richardson (1997), the recommended dietary allowance (RDA) for infants is 16 mg/100 g and none of the fermented products met the recommended dietary allowance (RDA) for vitamin B_3_ (niacin). Vitamin B_3_ is a part of the coenzyme, nicotinamide adenine dinucleotide (NAD) or its phosphate form, NADP that plays a key role in energy production and in the synthesis of fatty acids and steroids. The coenzyme is also involved in DNA replication, repair, and cell differentiation. Deficiency of this very important vitamin leads to pellagra (Institute of Medicine (US) Standing Committee on the Scientific Evaluation of Dietary Reference Intakes, 1998). The observed increase in the vitamin B concentration with fortification is in agreement with the report of Agunbiade, Bello, & Adeyemi, 2002; that observed increase in vitamin B concentration of bambara fortified maize sorghum mix.

### 3.6 Changes in amino acid profile

#### 3.6.1 Profile of tryptophan, isoleucine, and methionine

The profile of tryptophan, isoleucine, and methionine of the different fermenting maize‐pigeon pea ogi blends is presented in Table 7. Analysis of variance showed that there was significant effect (*p *≤* *.05) of fortification on the profile of the above‐mentioned amino acids of the different fermentation set‐ups.

**Table 7:** Profile of tryptophan, isoleucine, and methionine (mg/g) of the different fermenting maize‐pigeon pea ogi blends

Time (h)		Tryptophan (mg/g)			Isoleucine (mg/g)			Methionine (mg/g)		
		100:0	70:30	60:40	100:0	70:30	60:40	100:0	70:30	60:40
Steeping phase	0	4.54 ± 0.02^c^	19.54 ± 0.02^b^	30.60 ± 0.03^a^	15.17 ± 0.01^c^	38.85 ± 0.02^b^	50.21 ± 0.01^a^	30.84 ± 0.01^a^	26.24 ± 0.00^b^	25.46 ± 0.02^c^
	24	6.95 ± 0.01^c^	24.33 ± 0.01^b^	35.58 ± 0.00^a^	19.95 ± 0.02^c^	41.47 ± 0.00^b^	51.33 ± 0.01^a^	34.45 ± 0.00^a^	31.87 ± 0.02^b^	29.43 ± 0.02^c^
	48	7.50 ± 0.00^c^	28.02 ± 0.01^b^	39.27 ± 0.01^a^	20.97 ± 0.01^c^	43.12 ± 0.00^b^	52.29 ± 0.02^a^	40.18 ± 0.01^a^	38.05 ± 0.01^b^	36.66 ± 0.01^c^
Souring phase	0	2.59 ± 0.01^c^	10.09 ± 0.00^b^	19.75 ± 0.02^a^	9.78 ± 0.01^c^	20.22 ± 0.02^b^	31.03 ± 0.00^a^	33.45 ± 0.02^a^	31.87 ± 0.00^b^	30.98 ± 0.01^c^
	24	3.06 ± 0.00^c^	15.33 ± 0.01^b^	19.98 ± 0.02^a^	16.26 ± 0.02^c^	38.33 ± 0.01^b^	43.54 ± 0.01^a^	47.92 ± 0.00^a^	45.86 ± 0.01^b^	41.35 ± 0.02^c^
	48	3.75 ± 0.00^c^	17.85 ± 0.01^b^	20.38 ± 0.01^a^	21.50 ± 0.00^c^	48.86 ± 0.01^b^	54.78 ± 0.02^a^	50.07 ± 0.01^a^	47.55 ± 0.02^b^	43.75 ± 0.00^c^

Values are mean ± standard deviation of triplicate determinations. Means on the same row with different sets of superscripts are statistically different (*p* ≤ .05).

At the beginning of fermentation (0 hr steeping), 60:40 maize‐pigeon pea ogi had the highest tryptophan content (30.60 mg/g), and 100:0 maize‐pigeon pea ogi had the lowest tryptophan content (4.54 mg/g). As fermentation progressed, the levels of tryptophan increased but a great reduction was noticed at the beginning of the souring process (0 hr souring), which increased afterward. At the end of fermentation (48 hr souring), 60:40 maize‐pigeon pea ogi had the highest tryptophan content (20.38 mg/g); followed by 70:30 maize‐pigeon pea ogi (17.85 mg/g); and 100:0 maize‐pigeon pea ogi had the least tryptophan content (3.75 mg/g).

The same trend was observed with the isoleucine content, as with the tryptophan content. At the beginning of fermentation (0 hr steeping), 60:40 maize‐pigeon pea ogi had the highest isoleucine content (50.21 mg/g), whereas set‐up 100:0 maize‐pigeon pea ogi had the least isoleucine content (4.54 mg/g). As fermentation progressed, the levels of isoleucine increased but a great reduction was noticed at the 0 hr souring period, which increased afterward. At the end of fermentation (48 hr souring), 60:40 maize‐pigeon pea ogi had the highest isoleucine content (54.78 mg/g); followed by 70:30 maize‐pigeon pea ogi (48.86 mg/g); whereas 100:0 maize‐pigeon pea ogi had the least isoleucine content (21.50 mg/g).

The methionine content from the beginning (0 hr steeping) to the end (48 hr souring) of fermentation ranged from 25.46 to 43.75 mg/g in 60:40 maize‐pigeon pea ogi; 26.24–47.55 mg/g in 70:30 maize‐pigeon pea ogi; and 30.84–50.07 mg/g in 100:0 maize‐pigeon pea ogi.

#### 3.6.2 Profile of phenylalanine, leucine, valine, and lysine

The profile of phenylalanine, leucine, valine, and lysine of the different fermenting maize‐pigeon pea ogi blends is presented in Table 8. The result showed that there was significant effect (*p *≤* *.05) of fortification on the profile of the above‐mentioned amino acids of the different fermentation set‐ups.

**Table 8:** Profile of phenylalanine, leucine, valine, and lysine (mg/g) of the different fermenting maize‐pigeon pea ogi blends

Time (h)		Phenylalanine (mg/g)			Leucine (mg/g)			Valine (mg/g)			Lysine (mg/g)		
		100:0	70:30	60:40	100:0	70:30	60:40	100:0	70:30	60:40	100:0	70:30	60:40
Steeping phase	0	39.76 ± 0.01^c^	45.38 ± 0.01^b^	58.49 ± 0.00^a^	26.70 ± 0.00^c^	35.06 ± 0.01^b^	41.45 ± 0.01^a^	20.54 ± 0.02^c^	37.47 ± 0.01^b^	39.91 ± 0.01^a^	11.85 ± 0.02^c^	25.25 ± 0.01^b^	45.44 ± 0.00^a^
	24	44.52 ± 0.02^c^	51.23 ± 0.00^b^	61.55 ± 0.01^a^	33.55 ± 0.02^c^	43.81 ± 0.01^b^	53.31 ± 0.02^a^	36.25 ± 0.02^c^	40.01 ± 0.01^b^	41.44 ± 0.02^a^	28.46 ± 0.00^c^	30.04 ± 0.02^b^	63.92 ± 0.02^a^
	48	44.56 ± 0.01^c^	63.57 ± 0.02^b^	78.55 ± 0.02^a^	51.24 ± 0.01^c^	61.05 ± 0.01^b^	63.31 ± 0.01^a^	39.03 ± 0.01^c^	46.62 ± 0.07^b^	48.71 ± 0.01^a^	39.21 ± 0.00^c^	40.44 ± 0.02^b^	67.64 ± 0.01^a^
Souring phase	0	21.48 ± 0.00^c^	40.81 ± 0.02^b^	50.85 ± 0.00^a^	31.48 ± 0.02^c^	43.73 ± 0.01^b^	50.25 ± 0.01^a^	28.35 ± 0.01^c^	32.89 ± 0.01^b^	40.05 ± 0.00^a^	18.53 ± 0.01^c^	30.67 ± 0.07^b^	44.78 ± 0.00^a^
	24	40.63 ± 0.01^c^	65.45 ± 0.01^b^	70.01 ± 0.01^a^	55.33 ± 0.01^c^	77.75 ± 0.00^b^	96.32 ± 0.01^a^	36.10 ± 0.01^c^	52.21 ± 0.01^b^	56.37 ± 0.02^a^	19.15 ± 0.0^c^	61.66 ± 0.01^b^	78.52 ± 0.02^c^
	48	48.51 ± 0.01^c^	74.55 ± 0.01^b^	86.23 ± 0.02^a^	76.61 ± 0.13^c^	95.76 ± 0.01^b^	109.55 ± 0.00^a^	39.55 ± 0.02^c^	59.59 ± 0.01^b^	68.29 ± 0.01^a^	19.98 ± 0.01^c^	70.82 ± 0.00^b^	93.95 ± 0.01^a^

Values are mean ± standard deviation of triplicate determinations. Means on the same row with different sets of superscripts are statistically different (*p* ≤ .05).

At the beginning of fermentation (0 hr steeping), the phenylalanine content of the different fermenting maize‐pigeon pea ogi blends ranged from 39.76 mg/g (100:0 maize‐pigeon pea ogi) to 58.49 mg/g (60:40 maize‐pigeon pea ogi) and these values increased as fermentation progressed. However, at the beginning of the souring process (0 hr souring), there was a drop in phenylalanine content of the different fermenting maize‐pigeon pea ogi blends from 44.56 mg/g to 21.48 mg/g in 100:0 maize‐pigeon pea ogi; 63.57 mg/g–40.81 mg/g in 70:30 maize‐pigeon pea ogi; and 78.55 mg/g–50.85 mg/g in 60:40 maize‐pigeon pea ogi. At the 24 hr souring period, an increase in the phenylalanine content was observed in all the different maize‐pigeon pea ogi blends and this was the case till the end of fermentation (48 hr souring).

The same trend was also observed for the leucine and valine contents of all the different maize‐pigeon pea ogi blends. The leucine content at the beginning of fermentation (0 hr steeping) ranged from 26.70 to 41.45 mg/g and the valine content, from 20.54 to 39.91 mg/g; with 60:40 maize‐pigeon pea ogi having the highest leucine and valine content. At the end of fermentation (48 hr souring), the leucine content ranged from 76.61 to 109.55 mg/g, and the valine content from 39.55 to 68.29 mg/g, with 60:40 maize‐pigeon pea ogi having the highest leucine and valine contents.

The lysine profile of 60:40 maize‐pigeon pea ogi remained the highest throughout the period of fermentation. At the beginning of fermentation (0 hr steeping), the lysine profile ranged from 11.85 mg/g (100:0 maize‐pigeon pea ogi) to 45.44 mg/g (60:40 maize‐pigeon pea ogi). The lysine content continued to increase until the beginning of souring (0 hr souring), where there was a marked drop in the lysine content. However, values began to increase as souring progressed. At 48 hr souring which marked the end of fermentation, 60:40 maize‐pigeon pea ogi had the highest lysine content (93.95 mg/g), whereas 100:0 19.98 mg/g had the least lysine content (19.98 mg/g).

Amino acids are the chemical building blocks that make up proteins and proteins provide the structure for all living things as proteins participate in the vital chemical processes that sustain life. This study based analysis on some essential amino acids in the fermented products including tryptophan, isoleucine, methionine, phenylalanine, leucine, valine, and most importantly lysine. The recommended requirement for tryptophan for infants is 8.5 mg/g, according to FAO/WHO (1998), as reported in Haque et al. (2013). In this study, it can be observed that all the fermented products met the recommended requirement for tryptophan except product 100:0 (3.75 mg/g) which was limiting. The recommended requirement for isoleucine for infants is 35 mg/g, according to FAO/WHO (1998), as reported in Haque et al., 2013. It can be observed that the fortified fermented products met the recommended requirement for isoleucine, but the unfortified product 100:0 (21.50 mg/g) was limiting.

According to FAO/WHO (1998), as reported in Haque et al., 2013; the recommended requirement for methionine for infants is 29 mg/g. In this study, it can be observed that all the fermented products (100:0–50.07 mg/g; 70:30–47.55 mg/g; 60:40–43.75 mg/g) met the recommended requirement for methionine. The recommended requirement for phenylalanine for infants is 63 mg/g, according to FAO/WHO (1998), as reported in Haque et al., 2013. It can be observed that all the fermented products in this study met the recommended requirement for phenylalanine except product 100:0 (48.51 mg/g) which was limiting.

According to FAO/WHO (1998), as reported in Haque et al., 2013; the recommended requirements for leucine and valine for infants are 80 mg/g and 47 mg/g, respectively. In this study, it can be observed that all the fermented products met the recommended requirement for leucine and valine except product 100:0 (76.61 mg/g and 39.55 mg/g) which were limiting. The recommended requirement for lysine for infants is 52 mg/g, according to FAO/WHO (1998), as reported in Haque et al. (2013). It can be observed that the fortified fermented products met the recommended requirement for lysine but the unfortified product 100:0 (19.98 mg/g) was limiting.

### 3.7 Determination of antinutritional content

Changes in phytate and tannin content (mg/100 g), and trypsin inhibitor activity (%) of the different fermenting maize‐pigeon pea ogi blends are presented in Table 9. There was a massive reduction in antinutritional content of all the fermentation set‐ups from the beginning to the end of fermentation. A one‐way analysis of variance showed that there was significant difference (*p *<* *.05) between the values of the different antinutrient parameters of the different fermenting maize‐pigeon pea ogi blends, although the unfortified product (100:0 maize‐pigeon pea ogi blend) had much lower values than the fortified products (60:40 and 70:30 maize‐pigeon pea ogi blends).

**Table 9:** Changes in antinutritional contents of the different fermenting maize‐pigeon pea ogi blends

Time (h)		Phytate (mg/100 g)			Tannin (mg/100 g)			Trypsin inhibitor activity (%)		
		100:0	70:30	60:40	100:0	70:30	60:40	100:0	70:30	60:40
Steeping phase	0	90.23 ± 0.01^c^	396.56 ± 0.00^b^	446.84 ± 0.00^a^	13.15 ± 0.01^c^	13.78 ± 0.00^b^	18.39 ± 0.00^a^	3.19 ± 0.01^c^	44.30 ± 0.01^b^	61.11 ± 0.01^a^
	24	76.13 ± 0.01^c^	137.93 ± 0.20^b^	281.61 ± 0.01^a^	7.48 ± 0.00^c^	7.19 ± 0.01^b^	10.15 ± 0.01^a^	1.92 ± 0.02^c^	38.36 ± 0.01^b^	55.56 ± 0.01^a^
	48	40.07 ± 0.01^c^	90.52 ± 0.00^b^	150.86 ± 0.01^a^	3.72 ± 0.01^c^	4.00 ± 0.01^b^	6.48 ± 0.01^a^	0.96 ± 0.01^c^	30.43 ± 0.01^b^	36.84 ± 0.01^a^
Souring phase	0	9.48 ± 0.00^c^	26.01 ± 0.01^b^	34.63 ± 0.00^a^	2.24 ± 0.02^c^	3.24 ± 0.00^b^	4.19 ± 0.00^a^	1.14 ± 0.01^c^	21.43 ± 0.02^b^	26.32 ± 0.01^a^
	24	6.15 ± 0.01^c^	16.38 ± 0.00^b^	28.45 ± 0.01^a^	0.73 ± 0.00^c^	0.84 ± 0.00^b^	0.90 ± 0.01^a^	0.36 ± 0.01^c^	15.56 ± 0.01^b^	19.05 ± 0.01^a^
	48	2.54 ± 0.00^c^	9.48 ± 0.10^b^	13.36 ± 0.01^a^	0.30 ± 0.00^c^	0.39 ± 0.01^b^	0.44 ± 0.01^a^	0.10 ± 0.00^c^	10.77 ± 0.01^b^	12.50 ± 0.01^a^

Values are mean ± standard deviation of triplicate determinations. Means on the same row with different sets of superscripts are statistically different (*p* ≤ .05).

The phytate content at the beginning of fermentation (0 hr steeping) was highest in 60:40 maize‐pigeon pea ogi (446.84 mg/100 g) and lowest in 100:0 maize‐pigeon pea ogi (90.23 mg/100 g). As fermentation progressed, the phytate content kept decreasing until 13.36 mg/100 g in 60:40 maize‐pigeon pea ogi and 2.54 mg/100 g in 100:0 maize‐pigeon pea ogi at the 48 hr souring speriod which marked the end of the fermentation process.

A reduction in tannin content was observed. At the beginning of fermentation (0 hr steeping), the tannin content was highest in 60:40 maize‐pigeon pea ogi (18.39 mg/100 g); followed by 70:30 maize‐pigeon pea ogi (13.78 mg/100 g); and lowest in 100:0 maize‐pigeon pea ogi (13.15 mg/100 g). As fermentation progressed, the tannin content kept decreasing until 2.96 mg/100 g in 60:40 maize‐pigeon pea ogi; 1.73 mg/100 g in 70:30 maize‐pigeon pea ogi; and 0.50 mg/100 g in 100:0 maize‐pigeon pea ogi at the 48 hr souring period which marked the end of the fermentation process.

There was a significant decrease (*p *≤* *.05) in trypsin inhibitor activity (TIA) of the different fermenting maize‐pigeon pea ogi blends. At the beginning of fermentation (0 hr steeping), TIA was highest in 60:40 maize‐pigeon pea ogi (61.11%); followed by 70:30 maize‐pigeon pea ogi (44.30%); and lowest in 100:0 maize‐pigeon pea ogi (3.19%). As fermentation progressed, the trypsin inhibitor activity kept decreasing until 12.50% in 60:40 maize‐pigeon pea ogi; 10.77% in 70:30 maize‐pigeon pea ogi; and 0.10% in 100:0 maize‐pigeon pea ogi, at the end of fermentation (48 hr souring).

Antinutrients have the capacity of decreasing the digestibility and palatability of protein because they form insoluble complexes with them (Mbata et al., 2009). Phytates are known to form complexes with iron, zinc, calcium, and magnesium making them less available and thus inadequate in food samples especially for children. Tannins are naturally occurring plant polyphenols. Their main characteristic is to bind and precipitate protein interfering with its digestion and absorption. It is known that 10–50 mg phytate per 100 g will not cause a negative effect on the absorption of zinc and iron (Pikuda & Ilelaboye, 2013). In this study, the phytate contents of the different fermentation products at the end of fermentation ranged from 2.54 to 13.36 mg/100 g, which is within the safe consumption range. The tannin content of all the different fermented products in this study are generally low, ranging from 0.3 mg/100 g to 0.44 mg/100 g and are far lower than the lethal dose of 0.7–0.9 mg/100 g (Pikuda & Ilelaboye, 2013). This study showed a reduction in the tannins and phytic acid content, and trypsin inhibitor activity with increased fermentation period. Wakil and Kazeem (2012) also observed reduction in polyphenol contents of cereal‐ soyabean blends as a result of malting and toasting, and fermented cereal‐legume blends, respectively. The reduction in the phytic acid content of formulated blends may be due to hydrolysis of phytate by the enzyme phytase into lower inositol phosphates which are believed to be activated during the fermentation process by organisms (yeasts) whose hydrolyzing ability is enhanced by fermentation (Egwin, Elem, & Egwuche, 2011; Wakil & Kazeem, 2012). Previous reports have shown that lactic acid fermentation can also reduce tannins and phytic acid content, and trypsin inhibitor activity in bread fermentation (Chaoui, Zibilske, & Ohno, 2003; Khetarpaul & Chauhan, 1990).

## 4 CONCLUSIONS

This study showed that the fortified maize: pigeon pea products met the recommended dietary allowance (RDA) for moisture, fat, ash, protein, calcium, phosphorus, magnesium, iron, and vitamins B_1_ and B_2_. The fortified maize: pigeon pea products showed a significant decrease in antinutrient contents as antinutrients decrease the digestibility and palatability of proteins by forming insoluble complexes with them. The antinutrient content of all the fortified maize: pigeon pea products were shown to be within the safe consumption range. The fortified maize: pigeon pea products also met the recommended dietary allowance (RDA) for all the amino acids analyzed in this study (tryptophan, lysine, methionine leucine, isoleucine, and valine). It can therefore be concluded that the fortified maize: pigeon pea products 60:40 and 70:30 are safe for use as weaning foods.
